# Singlet oxygen production in *Chlamydomonas reinhardtii* under heat stress

**DOI:** 10.1038/srep20094

**Published:** 2016-02-01

**Authors:** Ankush Prasad, Ursula Ferretti, Michaela Sedlářová, Pavel Pospíšil

**Affiliations:** 1 Department of Biophysics, Centre of the Region Haná for Biotechnological and Agricultural Research, Faculty of Science, Palacký University, Šlechtitelů 27, 783 71 Olomouc, Czech Republic; 2 Department of Botany, Faculty of Science, Palacký University, Šlechtitelů 27, 783 71 Olomouc, Czech Republic

## Abstract

In the current study, singlet oxygen formation by lipid peroxidation induced by heat stress (40 °C) was studied *in vivo* in unicellular green alga *Chlamydomonas reinhardtii.* Primary and secondary oxidation products of lipid peroxidation, hydroperoxide and malondialdehyde, were generated under heat stress as detected using swallow-tailed perylene derivative fluorescence monitored by confocal laser scanning microscopy and high performance liquid chromatography, respectively. Lipid peroxidation was initiated by enzymatic reaction as inhibition of lipoxygenase by catechol and caffeic acid prevented hydroperoxide formation. Ultra-weak photon emission showed formation of electronically excited species such as triplet excited carbonyl, which, upon transfer of excitation energy, leads to the formation of either singlet excited chlorophyll or singlet oxygen. Alternatively, singlet oxygen is formed by direct decomposition of hydroperoxide via Russell mechanisms. Formation of singlet oxygen was evidenced by the nitroxyl radical 2,2,6,6-tetramethylpiperidine-1-oxyl detected by electron paramagnetic resonance spin-trapping spectroscopy and the imaging of green fluorescence of singlet oxygen sensor green detected by confocal laser scanning microscopy. Suppression of singlet oxygen formation by lipoxygenase inhibitors indicates that singlet oxygen may be formed via enzymatic lipid peroxidation initiated by lipoxygenase.

Reactive oxygen species (ROS) are formed by activation of non-reactive molecular oxygen during photosynthetic light reaction in chloroplasts, cellular respiration in mitochondria and defence against microorganisms in phagocyte plasma membrane^1^. The activation of molecular oxygen occurs either by an electron transport reaction known to form superoxide anion radical (O_2_^•−^), hydrogen peroxide (H_2_O_2_), hydroxyl radical (HO^•^) or by energy transfer reaction known to form singlet oxygen (^1^O_2_). Under circumstances when the formation of ROS exceeds the antioxidant capacity of the system, the equilibrium between production and scavenging is disturbed and the dangerous ROS induces damage to biomolecules, such are lipids, proteins and nucleic acids^1^.

Oxidation of lipids, known as lipid peroxidation, is initiated both by the non-enzymatic reactions involving the oxidation of lipids by ROS or by enzymatic reactions comprising oxidation of lipid mediated by enzymes such as lipoxygenase^2^. In the non-enzymatic reaction pathway, the lipid peroxidation is initiated either by radical ROS comprising HO^•^, generated by the Fenton reaction^1^,^3^, or by non-radical ROS involving ^1^O_2_ formed by the Type II photosensitisation reaction^4^,^5^,^6^. The initiation of lipid peroxidation by HO^•^ involves abstraction of weakly bonded hydrogen from polyunsaturated fatty acids known to form alkyl radical (L^•^) which in the presence of molecular oxygen, forms peroxyl radical (LOO^•^)^1^. Hydrogen abstraction from another polyunsaturated fatty acid by ROO^•^ forms hydroperoxides (LOOH)^7^. The initiation of lipid peroxidation by ^1^O_2_ involves the cycloaddition of ^1^O_2_ to polyunsaturated fatty acids that forms LOOH. In the enzymatic reaction pathway, lipid peroxidation is initiated by lipoxygenase known to exhibit dioxygenase activity. In this reaction, the ferric non-heme iron catalyzes the initial hydrogen abstraction forming ferrous non-heme iron and R^•^. The insertion of O_2_ at the C-atom of the polyunsaturated fatty acid known to form LOO^•^ is followed by reduction of ferrous non-heme iron and protonation of LOO^•^ to LOOH. Under reducing conditions such as reduced free and bound metals, LOOH is reduced to alkoxyl radical (LO^•^) which might further cause hydrogen abstraction from nearby located polyunsaturated fatty acids.

Several lines of evidence have been provided that indicates that lipid peroxidation is associated with formation of electronically excited species^8^,^9^. Decomposition of LOOH into peroxyl radical was proposed as a potential source of electronically excited species in biological systems^10^,^11^,^12^,^13^,^14^. In this reaction, LOOH is oxidized to LOO^•^ under oxidizing conditions such are oxidized transition metals, ferric heme iron of cytochrome c, peroxynitrite, chloroperoxide, and hypochlorous acid. Peroxyl radical might either undergoes cyclization to dioxetane or recombines to tetroxide^11^,^15^,^16^,^17^,^18^,^19^. These high energy intermediates decompose to triplet excited carbonyls (^3^L = O^*^) which might transfer triplet energy either to pigments forming excited pigments or molecular oxygen forming ^1^O_2_^10^,^20^,^21^,^22^,^23^,^24^. In addition, tetroxide might decompose directly to ^1^O_2_ by Russell mechanism^25^.

Under the environmental conditions, photosynthetic organisms such as cyanobacteria, algae and plants are exposed to various abiotic and biotic stress factors. Heat stress is a major environmental stress that is known to be involved in lipid peroxidation in photosynthetic organisms^26^,^27^,^28^. Extensive lipid peroxidation under heat stress was shown to be promoted by the enhancement in polyunsaturation of fatty acid in which hydrogen abstraction from the carbon next to double bond is energetically more feasible^29^,^30^. In agreement with this proposal, experimental data from many models indicate that the exposure of photosynthetic organisms to heat stress leads to the formation of lipid peroxidation secondary product, malondialdehyde (MDA), as detected by TBARS assay^31^,^32^,^33^,^34^. *In vitro*, it has been shown that exposure to heat stress results in the formation of the MDA-(TBA)_2_ adduct both in thylakoid membranes and PSII membranes isolated from higher plants^31^,^32^,^33^. *In vivo*, it has been demonstrated that exposure of leaf and root segments of *Phalaenopsis* to 40 °C enhanced lipoxygenase activity and MDA formation^31^,^32^,^33^,^34^.

Moreover, experimental evidence supports *in vitro*^1^O_2_ formation in chloroplasts, thylakoid and PSII membranes exposed to heat stress^31^,^35^,^36^. Using EPR spin-trapping spectroscopy, Hideg and Vass (1993) demonstrated that exposure of mung bean chloroplasts and etiolated thylakoid membranes to a non-physiological high temperature of 75 °C results in ^1^O_2_ formation. The authors tentatively attributed ^1^O_2_ formation to lipid peroxidation, although no experimental data were presented in this study. Later, it was shown that exposure of PSII membranes to 47 °C is accompanied by ^1^O_2_ formation^35^. The observation that ^1^O_2_ formation was unaffected either by catalase (known to decompose hydrogen peroxide to water and molecular oxygen) or mannitol as HO^•^ scavenger revealed that HO^•^ formed by incomplete water oxidation on PSII electron donor side unlikely initiated lipid peroxidation. Similarly, ^1^O_2_ formation was observed when spinach thylakoid and PSII membranes were exposed to heat stress at the temperature of 40 °C^31^. The authors demonstrated that the amount of MDA estimated spectroscopically by detection of the MDA-(TBA)_2_ adduct was formed in parallel to ^1^O_2_ formation. Based on the observation that the Q_B_ site on the PSII electron acceptor side was primarily damaged by heat stress, the authors proposed that ^1^O_2_ is formed near the Q_B_ site. In spite of the fact that ^1^O_2_ formation was demonstrated in chloroplast, thylakoid and PSII membranes, experimental evidence on ^1^O_2_ formation *in-vivo* has not yet been provided.

Our current study provides *in vivo* evidence that ^1^O_2_ is formed in the unicellular green alga *Chlamydomonas reinhardtii*. It is demonstrated herein that exposure of *Chlamydomonas* cells to heat stress (40 °C) results in the formation of 1) LOOH, the primary product of lipid peroxidation, as monitored by swallow-tailed perylene derivative (Spy-LHP) fluorescence as detected by confocal laser scanning microscopy, 2) MDA, the secondary product of lipid peroxidation, as monitored by HPLC detection of MDA-DNPH adduct, 3) ^3^L = O^*^ as measured by ultra-weak photon emission and 4) ^1^O_2_ localized by fluorescence of Singlet Oxygen Sensor Green (SOSG) visualized by confocal laser scanning microscopy and measured by electron paramagnetic resonance (EPR) spin-trapping spectroscopy utilising the oxidation of lipophilic diamagnetic 2,2,6,6-tetramethylpiperidine (TEMP). Attempts have been made to discuss the mechanism of ^1^O_2_ formation via lipid peroxidation initiated by enzymatic reaction catalysed by lipoxygenase.

## Results

### Hydroperoxide imaging using confocal laser scanning microscopy

**Figure 1:**
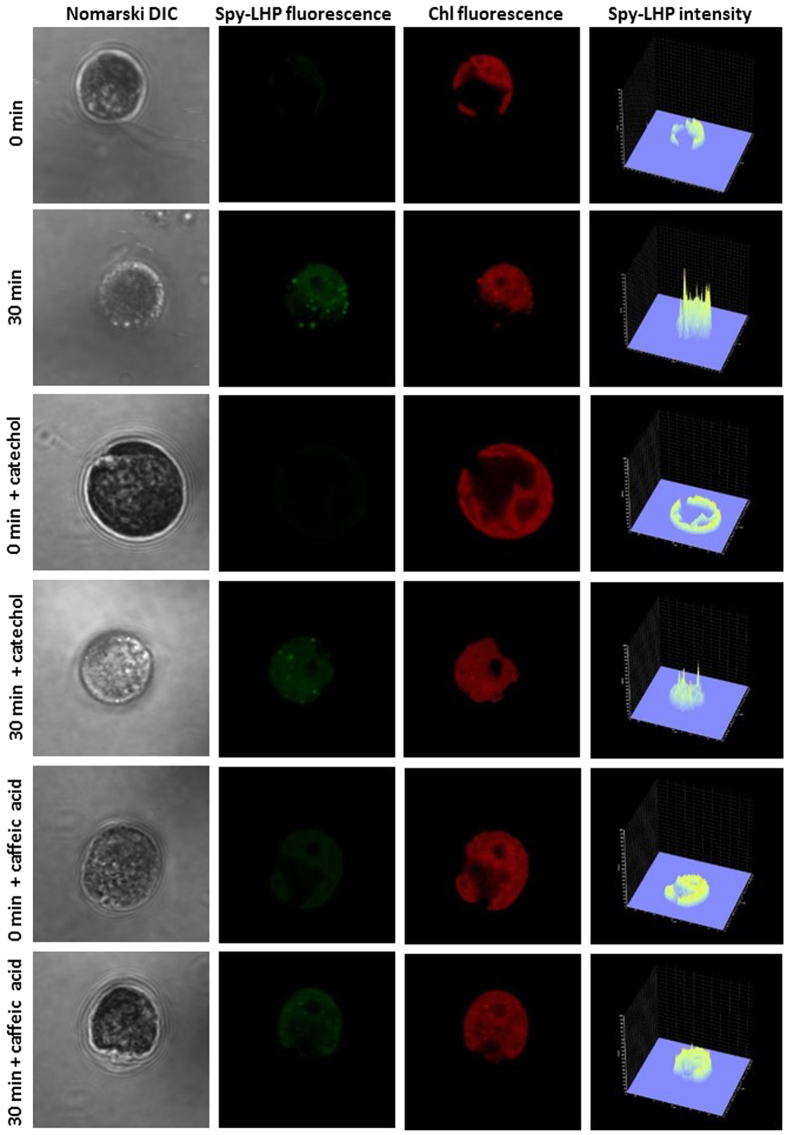
Detection of hydroperoxide in *Chlamydomonas* cells by laser confocal scanning microscopy. The formation of LOOH was measured in non-heated and heated *Chlamydomonas* cells in the presence and the absence of catechol and caffeic acid using fluorescent probe Spy-LHPOx. Heated cells were treated for 30 min in a water bath at 40 °C under dark. The images represent from left to right: Nomarski DIC, Spy-LHPOx fluorescence, chlorophyll fluorescence and integral distribution of Spy-LHPOx signal intensity (0–4096) in the 12-bit microphotographs.

The formation of LOOH in C*hlamydomonas* cells exposed to 40 °C was monitored using a fluorescent probe Spy-LHP detected by laser confocal scanning microscopy. Spy-LHP is known to react with LOOH leading to the formation of its oxidized derivative, Spy-LHPOx which provides a strong fluorescence at 535 nm. Figure 1 shows Nomarski DIC, Spy-LHPOx fluorescence, chlorophyll fluorescence and integral distribution of Spy-LHPOx fluorescence intensity measured in *Chlamydomonas* cells. Whereas no Spy-LHPOx fluorescence was detected in non-heated C*hlamydomonas* cells, pronounced Spy-LHPOx fluorescence was observed in heated C*hlamydomonas* cells exposed to 40 °C. The observation that localisation of chlorophyll fluorescence corresponds to localisation of Spy-LHPOx fluorescence indicates that LOOH is formed mainly in chloroplasts. In addition to this, bright spots of Spy-LHPOx fluorescence were observed which can be attributed to small-sized organelles such as vacuoles which usually are found in *Chlamydomonas* cells under various stress conditions. The integral distribution of Spy-LHPOx fluorescence intensity shows that Spy-LHPOx fluorescence in heated *Chlamydomonas* cells is enhanced by about 4 times as compared to non-heated *Chlamydomonas* cells. These results reveal that exposure of C*hlamydomonas* cells to heat stress leads to the formation of LOOH.

To test whether lipid peroxidation is initiated by non-enzymatic (ROS) or enzymatic (lipoxygenase) reactions, the effect of lipoxygenase inhibitors such as catechol and caffeic acid on LOOH formation was measured in heated C*hlamydomonas* cells (Fig. 1). Catechol and caffeic acid are known to inhibit lipoxygenase by binding to non-heme iron of enzyme and enzyme-substrate complex, respectively^37^,^38^. In non-heated *Chlamydomonas* cells, no effect of catechol on Spy-LHPOx fluorescence was observed. Interestingly, catechol suppressed significantly Spy-LHPOx fluorescence in heated *Chlamydomonas* cells as compared to heated cells measured in the absence of catechol. Similarly, no effect of caffeic acid on Spy-LHPOx fluorescence was observed in non-heated *Chlamydomonas* cell while Spy-LHPOx fluorescence was pronouncedly suppressed by caffeic acid in heated *Chlamydomonas* cells. The integral distribution of Spy-LHPOx fluorescence showed that in the presence of catechol and caffeic acid, Spy-LHPOx fluorescence was suppressed by approximately 40% and 70%, respectively, in heated *Chlamydomonas* cell. These results revealed that the inhibition of lipoxygenase by catechol and caffeic acid partially prevents LOOH formation.

### Quantification of hydroperoxides using ferrous oxidation-xylenol orange assay

**Figure 2:**
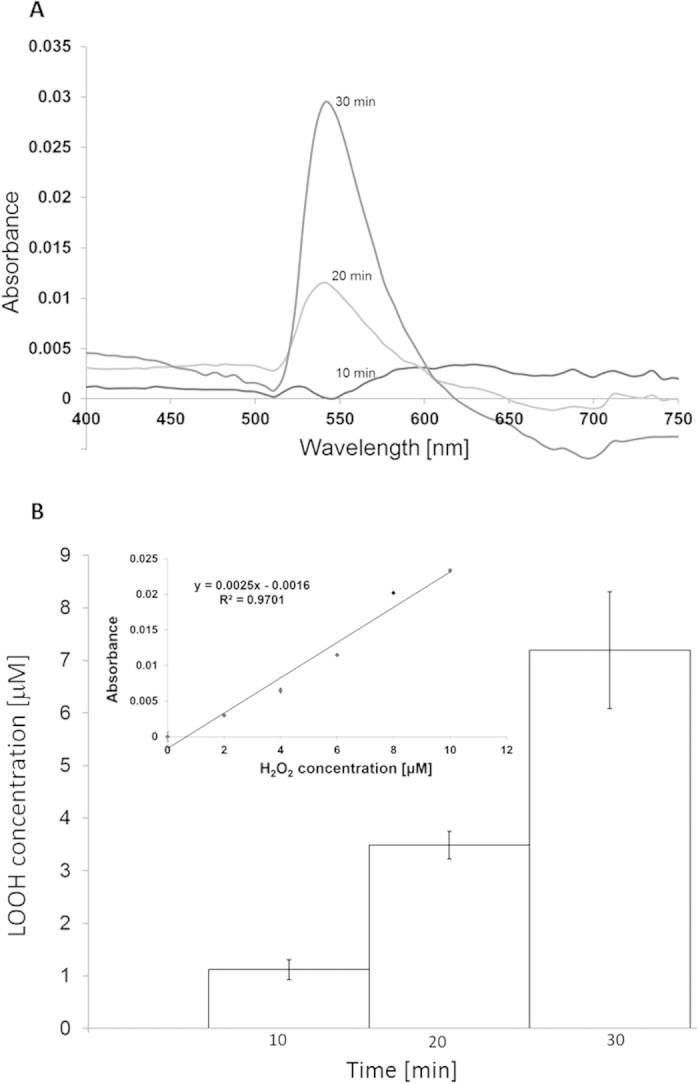
Quantification of hydroperoxide formation in *Chlamydomonas* cells ferrous oxidation-xylenol orange assay. In (**A**) absorption spectra of FOX reagent with *Chlamydomonas* cells heated for 10, 20 and 30 min measured in the spectral ranges of 400–750 nm. In (**B**) the concentrations of LOOH was established from calibration curve (equation y  =  0.0025x−0.0016) were as following: 1.12 ± 0.19 μmol (10 min), 3.49 ± 0.26 μmol (20 min) and 7.19 ± 1.11 μmol (30 min). The coefficient of determination R^2^ was determined as 0.9701. Insert shows calibration curve of FOX assay obtained using H_2_O_2_ as substrate.

To quantify LOOH formation in *Chlamydomonas* cells, ferrous oxidation-xylenol orange (FOX) colorimetric assay was used. In this method, the oxidation of ferrous to ferric irons by LOOH occurs with the subsequent binding of ferric irons to the dye xylenol orange, causing changes in its colour from yellow to red. Figure 2A shows FOX absorption spectra obtained as difference of spectra observed in heated and non-heated *Chlamydomonas* cells. The observation that absorption at 560 nm increased with a period of heat treatment shows gradual LOOH formation. The observation that catalase has no significant effect on absorption at 560 nm (data not shown) reveals that absorption changes are solely related to the LOOH formation with no contribution of H_2_O_2_. For determination of LOOH concentration formed in heated cells, the calibration curve was measured by FOX assay with H_2_O_2_ as the substrate (Fig. 2B, insert). The concentration of LOOH formed in heated *Chlamydomonas* cells is in the concentration range of several units of micromoles (Fig. 2B).

### Determination of the MDA-DNPH adduct using HPLC

**Figure 3:**
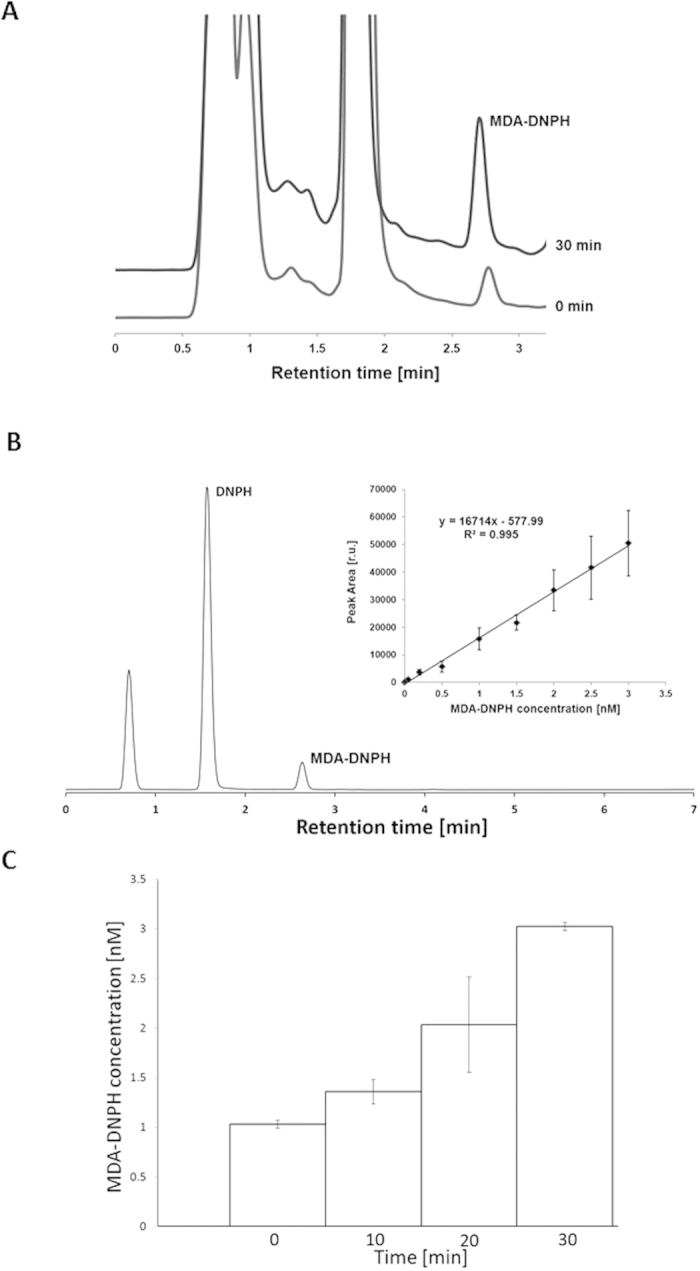
Detection of malondialdehyde by HPLC analysis in *Chlamydomonas* cells. The chromatogram of MDA-DNPH adduct in *Chlamydomonas* cells (**A**); MDA-DNPH adduct from standard TEP (**B**) and determination of MDA-DNPH adduct concentrations under heat stress (**C**). In A, chromatogram of MDA-DNPH adduct is shown in control (0 min) and heated (30 min) *Chlamydomonas* cells. Representative chromatograms were obtained as average of 3 chromatograms. In (**B**) chromatogram of MDA-DNPH adduct from MDA standard with a retention time of 2.75 min. The insert shows the dependence of average peak area (n = 5, ±SD) on the concentration of MDA-DNPH adduct from MDA standard TEP. In (**C**) the concentrations of MDA-DNPH adduct established from calibration curve (y = 16714x–577.99) were as following: 1.03 ± 0.04 nmol (0 min), 1.36 ± 0.12 nmol (10 min), 2.03 ± 0.48 nmol (20 min) and 3.02 ± 0.04 nmol (30 min), (n = 3, ±SD). The coefficient of determination R^2^ was determined as 0.995.

To examine the level of lipid peroxidation in *Chlamydomonas* cells exposed to heat stress, a secondary product of lipid peroxidation MDA was detected using isocratic reversed-phase HPLC separation of MDA-DNPH adduct. Figure 3A shows a chromatogram of the MDA-DNPH adduct measured in the *Chlamydomonas* cells treated at 40 °C for 0 min and 30 min. The MDA-DNPH adduct peak was observed at the retention time 2.75 min. To confirm the retention time of the MDA-DNPH adduct observed in *Chlamydomonas* cells, chromatogram of MDA standard 1,1,3,3-tetrahydroxypropane (TEP) was measured (Fig. 3B). To determine the concentration of MDA-DNPH adduct observed in heated cells, the calibration curve was established by plotting the peak area at 310 nm for various MDA-DNPH adduct concentrations obtained from MDA standard TEP (Fig. 3B, insert). Figure 3C shows that the concentration of MDA-DNPH adduct formed in heated *Chlamydomonas* cells is in the concentration range of several units of nanomoles.

### Detection of triplet excited carbonyl using ultra-weak photon emission

**Figure 4:**
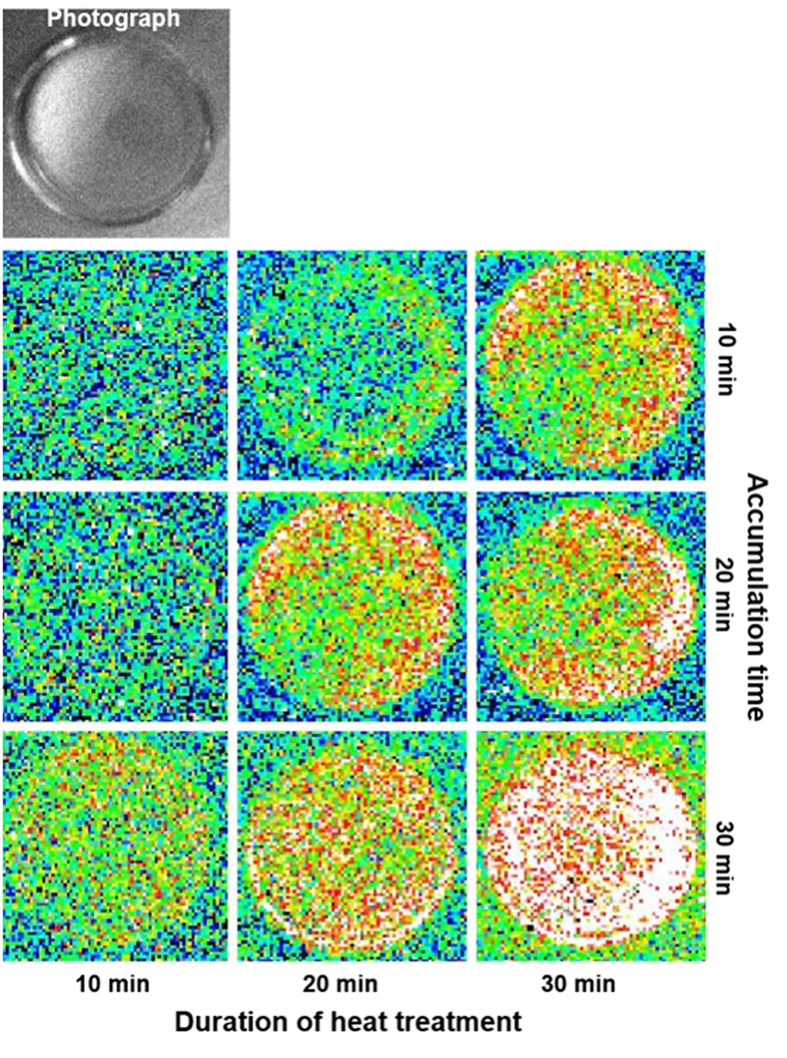
Two-dimensional imaging of ultra-weak photon emission from *Chlamydomonas* cells. Two-dimensional imaging of the spontaneous and induced ultra-weak photon emission from the *Chlamydomonas* cells was measured using highly sensitive CCD camera. The images and photographs of ultra-weak photon emission from the heated *Chlamydomonas* cells at 40 °C for 10, 20 and 30 min (X-axis). Ultra-weak photon emission imaging was measured with an accumulation time of 10, 20 and 30 min (Y-axis).

**Figure 5:**
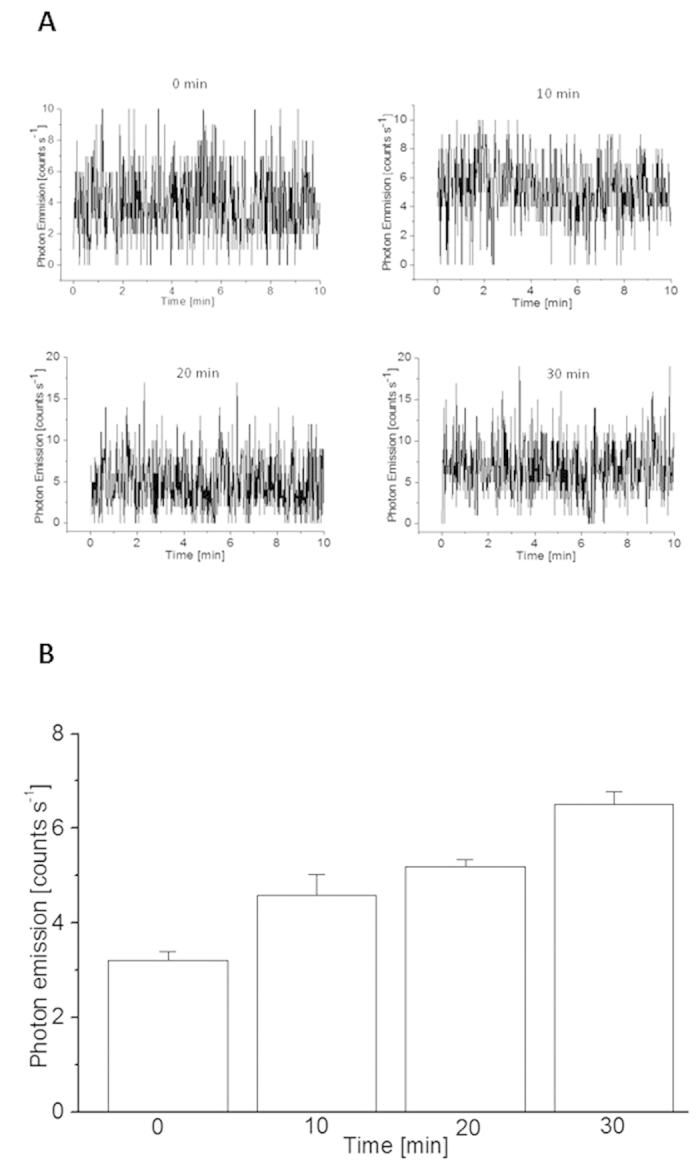
One-dimensional ultra-weak photon emission from *Chlamydomonas* cells. One-dimensional spontaneous and heat-induced ultra-weak photon emission were measured from *Chlamydomonas* cells using low-noise PMT. In (**A**) ultra-weak photon emission was measured in spontaneous and in heated *Chlamydomonas* cells at 40 °C for 0, 10, 20 and 30 min. In (**B**) mean photon emission intensity were measured in non-heated and heated *Chlamydomonas* cells. Heat treatment was done at 40 °C for 0, 10, 20 and 30 min. The presented data are expressed as the mean value and the standard deviation of at least three measurements (mean ± SD, n = 3).

To monitor the formation of ^3^L = O^*^ during lipid peroxidation, two-dimensional ultra-weak photon emission was measured in the *Chlamydomonas* cell using a highly sensitive charge coupled device (CCD) camera. Figure 4 shows the effect of heat treatment on the ultra-weak photon emission measured with accumulation time of 10, 20 and 30 min in the *Chlamydomonas* cells treated at 40 °C for 10, 20 and 30 min. When the cells were treated at 40 °C for a different time interval, photon emission was enhanced in proportion to the heat treatment period. To quantify ultra-weak photon emission from the cells, one-dimensional ultra-weak photon emission was measured using a low-noise PMT. Figure 5A shows that the photon emission in heated cells was found to be highest after 30 min, while the lowest photon emission was observed in cells without heat treatment. The photon emission in heated C*hlamydomonas* cells was found to be approximately enhanced by 30%, 40% and 50% in 10, 20 and 30 min of heat treatment, respectively compared to non-heated C*hlamydomonas* cells (Fig. 5B). These results indicates that exposure of C*hlamydomonas* cells to 40 °C results in the formation of ^3^L = O^*^.

### Singlet oxygen imaging using confocal laser scanning microscopy

**Figure 6:**
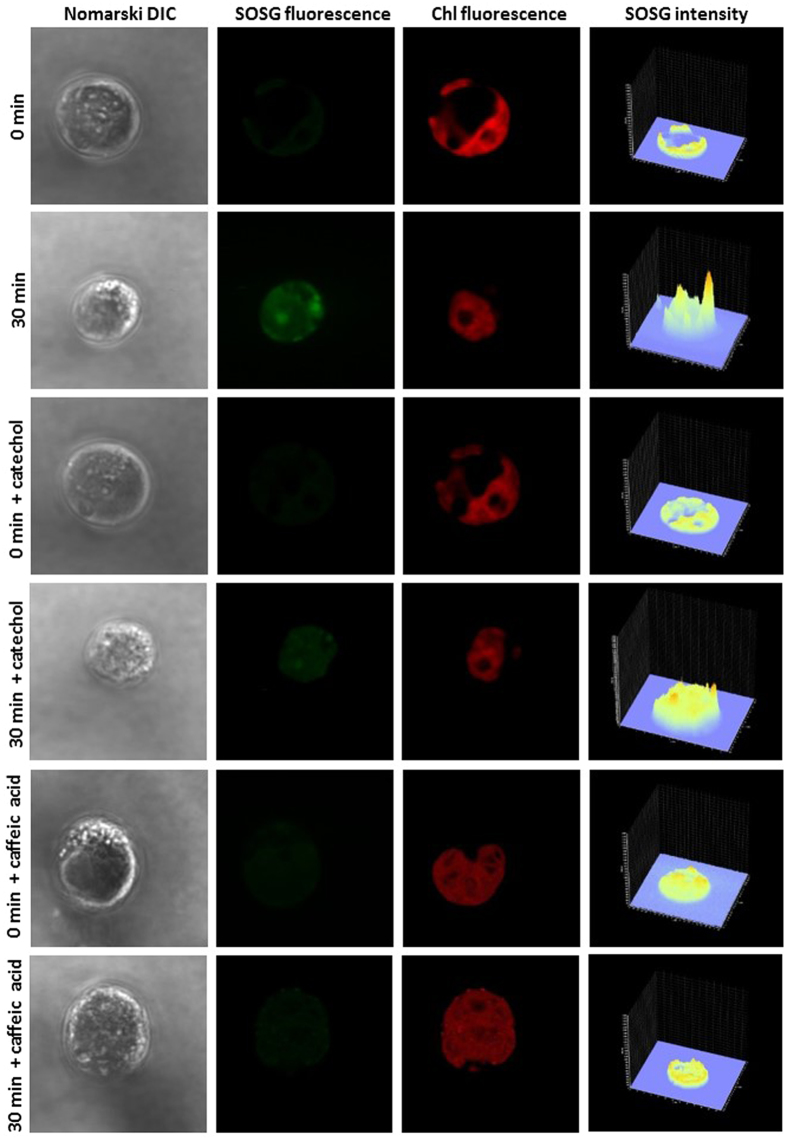
Detection of singlet oxygen in *Chlamydomonas* cells by laser confocal scanning microscopy. The formation of ^1^O_2_ was measured in non-heated and heated *Chlamydomonas* cells in the presence and the absence of catechol and caffeic acid using fluorescent probe SOSG. Heated cells were treated for 30 min in a water bath at 40 °C under dark. The images represent from left to right: Nomarski DIC, SOSG fluorescence, chlorophyll fluorescence and integral distribution of SOSG signal intensity (0–4096) in the 12-bit microphotographs.

To visualise ^1^O_2_ formation in the heated C*hlamydomonas* cells, ^1^O_2_ imaging was performed using the fluorescent probe SOSG as detected by laser confocal scanning microscopy. Figure 6 shows the Nomarski DIC images, the SOSG fluorescence, the chlorophyll fluorescence and the integral distribution of SOSG fluorescence intensity measured in *Chlamydomonas* cells. C*hlamydomonas* cells incubated with SOSG at room temperature exhibited very low SOSG fluorescence, whereas cells exposed to 40 °C emitted strong SOSG fluorescence representing ^1^O_2_ formation. SOSG fluorescence measured in multiple number of C*hlamydomonas* cells showed that distribution of ^1^O_2_ is not uniform among the cells (Supplementary data 1). The integral distribution of SOSG fluorescence intensity shows that SOSG fluorescence in heated *Chlamydomonas* cells is enhanced by about 6 times as compared to non-heated *Chlamydomonas* cells.

To localise ^1^O_2_ formation in heated C*hlamydomonas* cells, Nomarski DIC (A), SOSG fluorescence (B) and chlorophyll fluorescence (C) channels were compared for a series of optical sections through samples (Supplementary data 2). The observation that localisation of SOSG fluorescence corresponds to chlorophyll fluorescence reveals that ^1^O_2_ is formed predominantly in chloroplasts (Supplementary data 2A). Nomarski DIC images and SOSG fluorescence measured in multiple layers of C*hlamydomonas* cells show that SOSG fluorescence from the cytoplasm, pyrenoid and vacuoles also partially contributes to the overall SOSG fluorescence, indicating that ^1^O_2_ formation in other part of protoplast cannot be completely ruled out (Supplementary data 2B).

The effect of lipoxygenase inhibitors, catechol and caffeic acid on ^1^O_2_ was measured in heated C*hlamydomonas* cells. Figure 6 shows the Nomarski DIC, the SOSG fluorescence and the chlorophyll fluorescence images measured in the presence of catechol and caffeic acid in non-heated and heated C*hlamydomonas* cells. No significant effect on the SOSG fluorescence in the presence of catechol was observed in non-heated *Chlamydomonas* cell whereas the SOSG fluorescence was found to be significantly suppressed in the presence of catechol in the heated *Chlamydomonas* cell. The integral distribution of the SOSG fluorescence intensity measured in heated *Chlamydomonas* cells shows that the SOSG fluorescence intensity in the presence of catechol was lowered by approximately 60% as compared to the SOSG fluorescence intensity in the absence of catechol. Similarly, the effect of caffeic acid was tested on ^1^O_2_ imaging in heated C*hlamydomonas* cells. In non-heated *Chlamydomonas* cell, no change in SOSG fluorescence was observed under the effect of caffeic acid while in heated *Chlamydomonas* cells caffeic acid reduced signal to values of unheated samples as shown by the integral distribution of signal intensity (Fig. 6). These results reveal that inhibition of lipoxygenase by catechol and caffeic acid prevents ^1^O_2_ formation.

### Quantification of singlet oxygen using EPR spin-trapping spectroscopy

**Figure 7:**
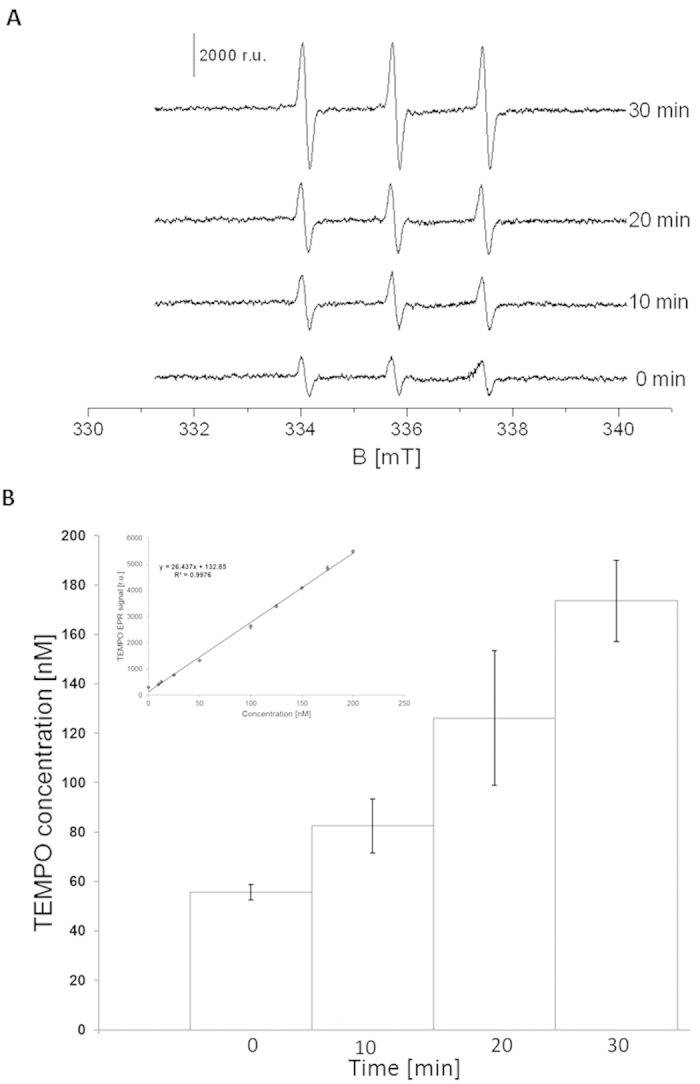
EPR spin-trapping detection of singlet oxygen formation from Chlamydomonas cells. Detection of ^1^O_2_ by EPR spin-trapping spectroscopy in *Chlamydomonas* cells. EPR spectra were detected after heat treatment for 0, 10, 20 and 30 min at 40 °C in the presence of 50 mM TEMP. In (**A**) shows time profile of TEMPO EPR spectra. The intensity of EPR signal was determined by measuring the relative height of central peak of the EPR absorption spectrum. Bar represents 2000 r.u. In (**B**) The presented data are expressed as the mean value and the standard deviation of at least three measurements (mean ± SD, n = 3). In (**B**) the concentrations of TEMPO was established from calibration curve (equation y = 26.437x + 132.85) were as following: 55.64 ± 3.04 nmol (0 min), 82.49 ± 10.89 nmol (10 min), 126.09 ± 27.28 nmol (20 min) and 173.55 ± 16.39 nmol (30 min). The coefficient of determination R^2^ was determined as 0.9976. Insert shows calibration curve of FOX assay obtained using H_2_O_2_ as substrate.

To quantify ^1^O_2_ formation in C*hlamydomonas* cells exposed to 40 °C, ^1^O_2_ was measured by EPR spin-trapping spectroscopy. The detection of ^1^O_2_ was accomplished using oxidation of lipophilic diamagnetic 2,2,6,6-tetramethylpiperidine (TEMP) by ^1^O_2_, which yields paramagnetic 2,2,6,6-tetramethylpiperidine-1-oxyl (TEMPO) (Fig. 7). In non-heated *Chlamydomonas* cells, the addition of TEMP shows a negligible TEMPO EPR signal caused by impurity of the spin trap. The treatment of *Chlamydomonas* cells to a temperature of 40 °C in the presence of TEMP resulted in the formation of the TEMPO EPR signal (Fig. 7A). To establish the concentration of ^1^O_2_ formed in heated cells, the calibration curve was obtained using TEMPO as a standard (Fig. 7B, insert). Figure 7B shows that the concentration of ^1^O_2_ formed in heated *Chlamydomonas* cells is in the concentration range of several tens to hundreds of nanomoles.

## Discussion

Several pieces of evidence have been provided that indicate that the increase in temperature is associated with lipid peroxidation^33^,^34^,^39^,^40^. In agreement with this evidence, we showed that the exposure of C*hlamydomonas* cells to 40 °C leads to the formation of LOOH (Fig. 1). The observation that lipoxygenase inhibitors (catechol and caffeic acid) significantly suppressed LOOH formation reveals that lipid peroxidation is initiated by lipoxygenase (Fig. 1). Quantification of LOOH formation using FOX assay showed that LOOH was formed in the concentration range of several units in micromoles (Fig. 2). In the propagation phase, the lipid peroxidation process propagates via the formation of LOOH formed upon reaction of LOO^∙^ with another lipid molecule. In termination, the cyclisation of LOO^∙^ is known to form cyclic endoperoxide, the decomposition of which leads to the formation of MDA. Our observation that the MDA-DNPH adduct detected by HPLC (Fig. 3) was enhanced in heated *Chlamydomonas* cells compared to non-heated cells reveals a higher degree of lipid peroxidation under heat stress.

Alternatively, the cyclisation of LOO^•^ is known to form cyclic dioxetane or the recombination of LOO^•^ forms linear tetroxide^18^,^20^,^21^. The decomposition of dioxetane or tetroxide results in the formation of ^3^L = O^*^^16^,^17^. Our recent results on the spectral analysis of linoleic acid induced ultra-weak photon emission from *Chlamydomonas* cells using band pass filters showed that the photon emission is in red region of the visible spectrum (> 600 nm), with a photon emission maximum at 680 nm indicating that the photon emission is predominantly from singlet excited chlorophylls. The photon emission in the blue-green region of the spectrum from ^3^L = O^*^ is quite negligible because the energy transfer from ^3^L = O^*^ to chlorophyll is more efficient than the photon emission. As singlet excited chlorophylls are formed solely by excitation energy transfer from ^3^L = O^*^ to chlorophylls^21^, ultra-weak photon emission might serve as an indirect indicator of ^3^L = O^*^ formation. In agreement with our observations, it has been previously demonstrated that exposure of spinach leaves and isolated chloroplasts to heat stress is accompanied by the photon emission from singlet excited chlorophyll^41^. The authors showed that photon emission is dominant in the wavelength range from 700–800 nm under heat treatment. The photon emission from both the samples was found to increase with the increase in temperature from 0 to 40 °C. The slow increase in photon emission in the temperature range of 0–25 °C can be because of initiation of metabolic processes; however, a steep rise in the temperature range of 25–45 °C can be related to the high activity of lipoxygenase present in leaves and isolated chloroplasts. Based on these considerations, the enhancement in ultra-weak photon emission observed in *Chlamydomonas* cells exposed to heat stress (Figs 4 and 5) indicates that ^3^L = O^*^ are formed in the heated *Chlamydomonas* cells during lipid peroxidation likely initiated by lipoxygenase.

The triplet-singlet excitation energy transfer from ^3^L = O* to molecular oxygen leads to the formation of ^1^O_2_. Alternatively, ^1^O_2_ is formed directly by decomposition of tetroxide to ^1^O_2_ via the Russell reaction^10^,^18^,^21^. Due to elimination of LOO^•^ by the antioxidant system in *Chlamydomonas* cells, the probability of recombination of two LOO^•^ is significantly low. Under these circumstances, ^1^O_2_ formation via decomposition of LOOOOL via the Russell reaction is less expected^11^,^42^,^43^. Based on these considerations, it seems to be likely that cyclisation of LOO^•^ to LOOL and its subsequent decomposition to ^3^L = O* is the probable reaction pathway for ^1^O_2_ formation. EPR spin-trapping spectroscopy showed that exposure of *Chlamydomonas* cells to 40 °C results in the formation of ^1^O_2_ (Fig. 7A). The observation that ^1^O_2_ formation linearly increases up to 30 min of heat treatment indicates continuous ^1^O_2_ formation with increase in duration of heat treatment (Fig. 7B). The imaging of ^1^O_2_ using the green fluorescence of SOSG detected by confocal laser scanning microscopy confirmed that exposure of *Chlamydomonas* cells to 40 °C results in the formation of ^1^O_2_ (Fig. 6). Based on the results obtained using green fluorescence of SOSG and chlorophyll fluorescence, it is evident that ^1^O_2_ formation occurs predominantly within chloroplast (Supplementary data 1); however, the contribution of other parts of a cell protoplast are very probable to contribute to the ^1^O_2_ pool in the heated C*hlamydomonas* cells (Supplementary data 2). The observation that inhibition of lipoxygenase by catechol partially prevented ^1^O_2_ formation indicates that the lipid peroxidation that leads to ^1^O_2_ formation is initiated by lipoxygenase (Fig. 6).

Differences in the localisation of LOOH (Fig. 1) and the ^1^O_2_ (Fig. 6 and Supplementary data 2) suggest that formation of both LOOH and ^1^O_2_ is localised predominantly in chloroplasts. Contrary to LOOH, ^1^O_2_ formation was also observed in pyrenoid, cytoplasm and vacuoles likely due to the short-distance diffusion of ^1^O_2_ from the site of its formation. These results provide experimental support for the correlation of lipid peroxidation and ^1^O_2_ formation.

## Methods

### *Chlamydomonas reinhardtii* growth conditions

Algae strain, *Chlamydomonas reinhardtii* (wild type: CC-002) was obtained from the Chlamydomonas Genetic Center (Duke University, Durham, NC, USA). The cells were cultivated in Tris-Acetate-Phosphate (TAP) medium in a continuous white light (100 μmol photons m^−2^ s^−1^) in Algaetron AG 230 (Photon Systems Instruments, Drásov, Czech Republic). The growth was achieved under permanently stirred condition using shaker (Orbital Shaker PSU-10i, Biosan, Riga, Latvia) to obtain constant CO_2_ concentration in the growing medium. The cells were studied at a density of approximately 7×10^7 ^cells ml^−1^ during the stationary growth phase. The cell density was determined by an automated cell counter (TC20 Automated Cell Counter, BioRad, Hercules, CA, U.S.A.).

### Heat treatment

The lipid peroxidation in *Chlamydomonas* cells were induced using heat stress. The cell were exposed solely to heat stress and any effect of light was prevented. Samples were treated for 10, 20 and 30 min at temperature of 40 °C using in a water bath (Julabo GmbH, Germany) in Eppendorf tubes.

### Confocal laser scanning microscopy

*In-vivo* imaging of LOOH and ^1^O_2_ was based on their reaction with a swallow-tailed perylene derivative (Spy-LHP) (Dojindo Molecular Technologies Inc. Rockville, MD, USA) and SOSG (Molecular Probes Inc., Eugene, OR, USA), respectively. *Chlamydomonas* cells were incubated either in the presence of 20 μM Spy-LHP or 50 μM SOSG in darkness for 30 min. To study an influence of temperature stress, samples were kept at room temperature or subjected to 40 °C. Immediately after staining, the cells were transferred to a fresh TAP medium and visualized by confocal laser scanning microscopy (Fluorview 1000 unit attached to IX80 microscope; Olympus Czech Group, Prague, Czech Republic). The excitation of both fluorochromes were achieved by a 488 nm line of an argon laser and signal was detected either by a 505–550 nm emission filter for LOOH or by a 505–525 nm emission filter for ^1^O_2_. Chlorophyll fluorescence from chloroplasts of *Chlamydomonas* cells was achieved by excitation with 543 nm helium-neon laser, and emission recorded with a 655–755 nm band filter. Cell morphology was visualized by transmitted light detection module with 405 nm excitation using a near ultraviolet (405 nm) diode laser and Nomarski DIC filters. The proper intensity of all lasers was set according to unstained samples at the beginning of each experiment (Sedlářová *et al.* 2011)^44^. Integral distribution of signal intensity (0–4096) in 12-bit microphotographs was evaluated by image analysis software FV10-ASW Viewer (Olympus).

### High performance liquid chromatography (HPLC)

Malondialdehyde, a product of lipid or protein oxidation was measured using HPLC. The isolation and derivatization of MDA using 2,4-dinitrophenylhydrazine (DNPH) was performed as described in Pilz *et al.* (2000) with some modifications^45^. After heat treatment, cells were centrifuged at 2000 x g for 10 min and the supernatant was removed. The pellet was resuspended in 200 μl of phosphate buffer saline (PBS, pH  =  7.5) and 100 μl 0.06% butylhydroxytoluene (BHT) dissolved in methanol. Using a sonicator for 90 s (Sonicator, Ultrasonic homogenizer Model 3000, Biologics Inc., Manassas, VA, U.S.A.), the *Chlamydomonas* cells were disrupted. This step was followed by centrifugation at 2000 x g for 10 min and 125 μl of supernatant was taken for following step. To achieve alkaline hydrolysis of protein bound MDA, 25 μl of 6 M aqueous sodium hydroxide was added to the samples and sample were treated in a 60 °C dry bath for 30 min (Thermo-Shaker TS100, Biosan, Riga, Latvia). To reach the precipitation of proteins in samples, 62.5 μl of 35% (v/v) perchloric acid was added to the sample, vortexed and centrifuged at 16000 x g for 10 min. 125 μl of supernatant was taken into a vial and resuspended in 1 μl of 50 mM DNPH dissolved in 50% sulphuric acid and treated in dark at room temperature for 30 min. DNPH bound to MDA to create a MDA-DNPH adduct. An amount of 25 μl was injected into the HPLC system (Alliance e 2695 HPLC System, Waters, Milford, MA, U.S.A.) and detected at 310 nm using UV/VIS detector. A Symmetry C18 (3.5 μm; 4.6 × 75 mm) Column (Waters, Milford, MA, U.S.A.) was used. The analysis was performed isocratically (1 ml/min at 35 °C) using mobile phase comprised of mixture of 25 mM trimethylamine (pH 3.5) and acetonitrile in the ratio 50:50 (v:v). To remove impurities from the column after every measurement, the column was rinsed by 100% methanol.

### Ferrous oxidation-xylenol orange assay

For the quantification of LOOH in *Chlamydomonas* cells, FOX assay (John M. DeLong *et al.* 2002) was used with minor modifications. FOX reagent was prepared by mixing 50 mM xylenol orange and 5 mM iron(II) sulfate heptahydrate in proportion 1:1. For the measurement 100 μl of sample (reference) and 2000 μl FOX reagent was used. FOX reagent was added to *Chlamydomonas* cells prior to heat treatment and absorption changes were measured 30 min after start of heat treatment to keep the total period constant. Hydroperoxide formation was monitored by following the absorbance changes at 560 nm using Olis RSM 1000 spectrometer (Olis Inc., Bogart, Georgia, USA).

### Ultra-weak photon emission measurement

*Chlamydomonas* cells in TAP medium (total volume of 2 ml) in a glass Petri dish (3 cm diameter) were used for ultra-weak photon emission measurements. For two-dimensional photon emission imaging, the photons were reflected by a mirror to the CCD camera positioned horizontally. For one-dimensional photon counting, the Petri dish was placed below the vertically positioned photomultiplier tube (PMT) at a distance of 3 cm. The cells were dark-adapted for 30 min to eliminate any interference by delayed luminescence.

Highly sensitive CCD camera VersArray 1300B (Princeton instruments, Trenton, NJ, USA) with spectral sensitivity in the range of 200–1000 nm restricted to 350–1000 nm due to the objective lens mounted and almost 30% quantum efficiency in the visible range of the spectrum was used for two-dimensional photon imaging. For reduction of dark current, CCD camera was cooled down to −104 °C using a liquid-nitrogen cooling system. The data correction was made by subtracting the background noise before every measurement. The measurement was done in the image format of 1340 × 1300 pixels with following CCD camera parameters: scan rate, 100 kHz; gain, 3; accumulation time, 30 min.

One-dimensional photon counting was done using low-noise photon counting unit C9744 (Hamamatsu Photonics K.K., Iwata city, Japan) with a spectral sensitivity in the range of 160–710 nm. For reduction of thermal electrons, PMT was cooled down to −30 °C using thermoelectric cooler C9143 (Hamamatsu Photonics, K.K., Iwata city, Japan). The PMT was kept vertically to minimize the dark counts to ~2 counts s^−1^ at −1150 mV. The photon emission measurements were done in the experimental darkroom (3 m × 1.5 m × 2.5 m) painted with black colour. The door in the experimental darkroom was protected completely with a black curtain to restrict any diffusion of light from external source.

### Electron paramagnetic resonance (EPR) spectroscopy

EPR spin-trapping spectroscopy was used to measure the ^1^O_2_ production. *Chlamydomonas* cells suspended in TAP media with 50 mM TEMP spin trap were heated at 40 °C and EPR spectra were recorded using an EPR spectrometer MiniScope MS400 (Magnettech GmbH, Berlin, Germany). To eliminate impurity TEMPO EPR signal, TEMP was purified twice by vacuum distillation. EPR conditions are as follows: microwave power, 10 mW; modulation amplitude, 1 G; modulation frequency, 100 kHz; sweep width, 100 G; scan rate, 1.62 G s^−1^.

## Additional Information

**How to cite this article**: Prasad, A. *et al.* Singlet oxygen production in *Chlamydomonas reinhardtii* under heat stress. *Sci. Rep.* **6**, 20094; doi: 10.1038/srep20094 (2016).

## Supplementary Material

Supplementary Information
