# Supplementary material for: Singlet oxygen production in Chlamydomonas reinhardtii under heat stress
Source: Sci Rep. 2016 Feb 1;6:20094. doi: 10.1038/srep20094 (PMC4757480; doi:10.1038/srep20094)
Supplement: Supplementary Information [file srep20094-s1.pdf]

Singlet oxygen production in *Chlamydomonas reinhardtii* under heat stress

Ankush Prasad, Ursula Ferretti, Michaela Sedlářová, Pavel Pospíšil

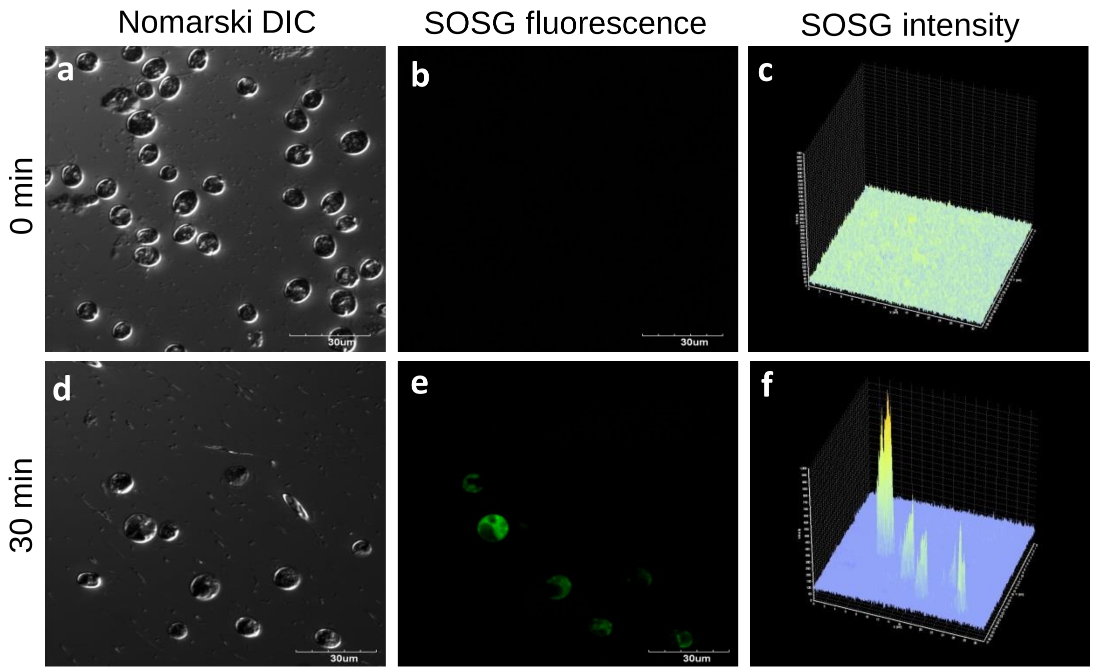

Supplementary data 1: Detection of singlet oxygen in multiple cells of Chlamydomonas by laser confocal scanning microscopy. *Chlamydomonas* cells at room temperature and heated at 40 °C during 30 min of SOSG staining as detected by confocal laser scanning microscope (λ_{ex}=488nm, λ_{em}=505-525nm). The images represent (from left to right) Nomarski DIC, SOSG fluorescence and distribution of SOSG intensity in and among different cells.

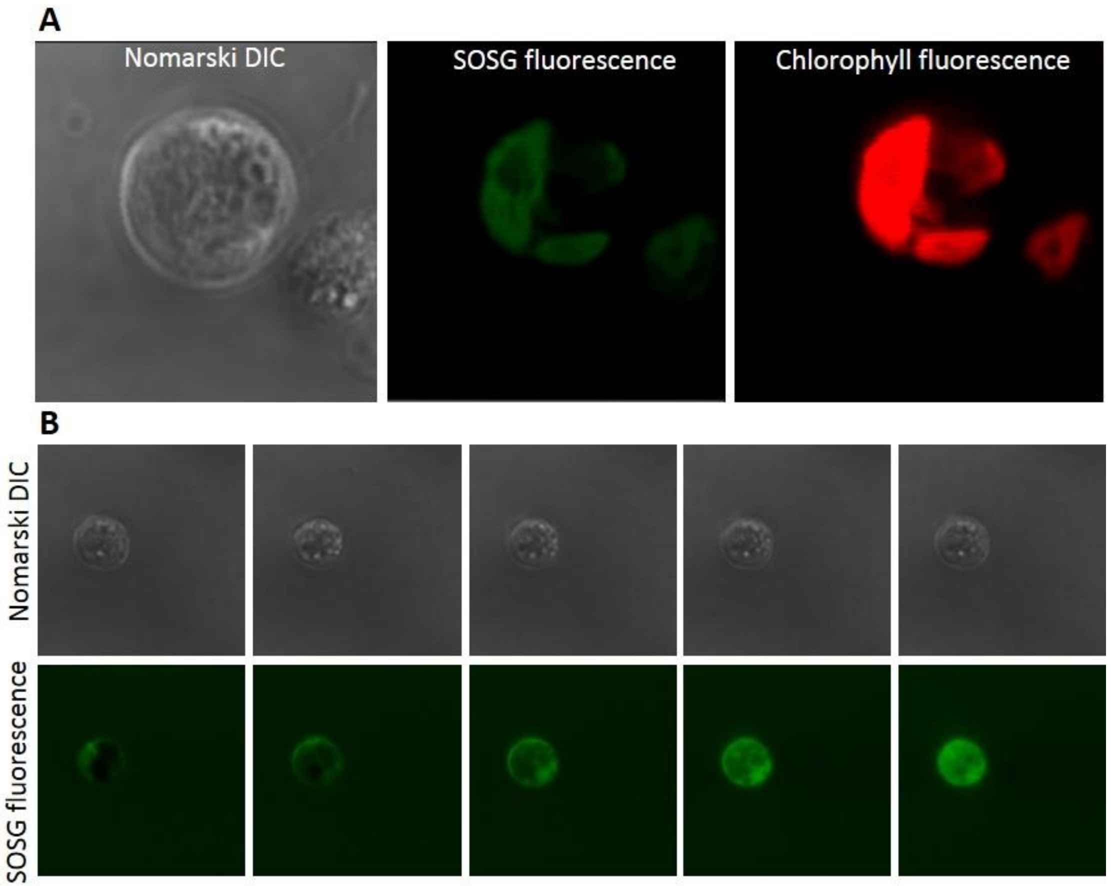

Supplementary data 2: Detection and localization of singlet oxygen formation in Chlamydomonas cells by laser confocal scanning microscopy (A); *Chlamydomonas* cells heated at 40 °C during 30 min of SOSG staining as detected by confocal laser scanning microscope (λ_{ex}=488nm, λ_{em}=505-525nm). The images represent (from left to right) Nomarski DIC, SOSG fluorescence and chlorophyll fluorescence distribution. (B); Localization of $^1O_2$ in heated (30 min) *Chlamydomonas* cells using SOSG fluorescence measured in the multiple layers of C*hlamydomonas* cells.
